# Regional gaps in the provision of inpatient rehabilitation services for the elderly in Israel: Results of a national survey

**DOI:** 10.1186/2045-4015-2-27

**Published:** 2013-07-23

**Authors:** Inbar Zucker, Irit Laxer, Iris Rasooli, Shulamit Han, Aaron Cohen, Tamar Shohat

**Affiliations:** 1 Israel Center of Disease Control, Ministry of Health, Jerusalem, Israel; 2 Geriatrics Department, Ministry of Health, Jerusalem, Israel

**Keywords:** Stroke, Hip fracture, Rehabilitation, Elderly, Health care disparities

## Abstract

**Background:**

Medical events, such as stroke, limb fractures, joint replacements and spinal injuries, can lead to acute functional disability at all ages and to chronic disability, especially among the elderly. Rehabilitation is, therefore, essential for the prevention of permanent disability among older individuals. There are international practice guidelines for stroke and hip fracture management, including recommendations that rehabilitation services be an integral part of the provision of treatment in either an inpatient setting or in the community. There are no organized data on provision of rehabilitation services in Israel or on the distribution of these services throughout the country. Such information would be of great assistance in designing these services where they are needed and in making changes in the existing ones where necessary.

**Methods:**

Patients aged 65 years or older with stroke or hip fracture were identified through one-day surveys conducted in 2009–2010 in all 26 acute care hospitals in Israel. Data on inpatient and ambulatory rehabilitation services were collected from discharge medical summaries, telephone interviews with the patients or their relatives and reports from the healthcare provider. The extent of rehabilitation services was described and the association between receipt of inpatient rehabilitation and the geographic district based on the patients’ listed address was examined in a multivariate analysis.

**Results:**

A total of 570 patients with stroke and 768 patients with hip fracture were identified and interviews were conducted in regards to 421 and 672 respectively. Out of the stroke patients 238(56.5%) received inpatient rehabilitation, 46(10.9%) received ambulatory rehabilitation treatment without inpatient phase and 137 (32.5%) received no rehabilitation. In fracture these rates were 494(73.5%), 96(14.3%) and 82(12.2%) respectively. Patients living in districts with lower availability of rehabilitation beds were less likely to receive inpatient rehabilitation after controlling for patient characteristics.

**Conclusions:**

Regional disparities in the provision of inpatient rehabilitation care for elderly after an acute episode of stroke or hip fracture were identified and could be partially attributed to the distribution of rehabilitation beds. These findings highlight the need to plan the rehabilitation resources based on the population needs and to routinely monitor the provision of these services.

## Background

Rehabilitation is essential following medical events that lead to an acute functional disability, such as stroke, limb fractures, joint replacements and spinal injuries [1]. The incidence of these events increases sharply with age [2-4], and the elderly are also more prone to become dependent as a result of a physical functional insult [5]. Therefore, the provision of proper rehabilitation services is of critical importance to the elderly population [1]. The provision of organized multidisciplinary post-acute rehabilitation care after stroke or hip fracture can reduce long-term mortality and institutionalization [6-9]. International practice guidelines for both stroke and hip fracture management recommend that rehabilitation services be provided as an integral part of the treatment plan in the acute and post-acute phases in order to minimize loss of function and prevent disability [7,10-14]. Post-acute rehabilitation treatment can be provided in either an inpatient setting or in the community. Regardless of the setting, in order to be effective rehabilitation services should be provided by a multidisciplinary coordinated team with sufficient intensity [8,9].

Israel has a limited number of rehabilitation day-centers that offer multidisciplinary treatment in an outpatient setting. Therefore, post-acute care usually starts in a specialized rehabilitation inpatient facility. Currently, there are 18 licensed wards for geriatric rehabilitation and ten for general rehabilitation, with a total of about 1500 beds for general and geriatric rehabilitation. This translates into about 2 beds per 1000 residents 65 year or older. In 2009, the Ministry of Health in collaboration with the Ministry for Senior Citizens launched a multidisciplinary initiative to improve and promote the medical rehabilitation services delivered to the elderly in Israel. The initiative encompassed the dissemination of practice guidelines to the acute care wards regarding the referral process for rehabilitation treatment [15] and the launching of a public campaign to increase patients‘ awareness of their rights, in addition to extending the Ministry‘s supervision of rehabilitation services. This initiative was accompanied by the collection of data on the extent of rehabilitation services provided to elderly patients following stroke or hip fracture in a database that could serve as a source for future re-evaluation. The aims of the present study were to characterize the provision of rehabilitation services to elderly individuals after undergoing a stroke or hip fracture, and to determine whether there were any disparities between geographic districts throughout the country. Our belief is that such information would be of great assistance in developing rehabilitation services according to regional needs and in making required changes in the existing ones.

## Methods

### Study population

The study population included patients aged 65 years or older who were hospitalized for acute stroke or hip fracture. The patients were identified during a series of 6 single-day surveys conducted in the neurology, internal medicine, orthopedic and geriatric wards in all of the 26 general hospitals in Israel. Data regarding rehabilitation was collected only for those who were discharged from the acute care after exclusion of in-hospital mortality.

### Data sources and study variables

Data on each study patient were collected from three sources. The first was the medical discharge letter which was used to extract data on demographic variables, the patient’s functional status prior to the hospitalization, co-morbidities, course of the acute event, in-hospital complications, and recommendations for rehabilitation treatment. Three variables were used to describe the patient’s functional status prior to the event: indoor mobility was dichotomized to mobile (independent or with assistance) versus wheel-chair dependent, general function was dichotomized to fully dependent versus independent or need some assistance and cognitive function was dichotomized to with or without dementia. The severity of stroke was assessed according to the NIH stroke scale (NIHSS) which has been noted to be a god predictor of patient outcomes [16,17]. NIHSS assessment was based on the neurologic examination at admission that was recorded in the hospital discharge summary.

The second source was a telephone interview with the patient, or with a close family member, which was conducted 4–6 months after the patient was discharged in order to collect information on any inpatient or ambulatory rehabilitation care that had been received. The contact details of a family member were taken from the hospital medical record. A caretaker was contacted only if the patient could not be interviewed. The interview started by asking the family member if he was familiar with the patients’ care, and if not he was asked to provide contact details for an appropriate caretaker. The third source was the administrative data on any inpatient rehabilitation that the patient received within the first four months after hospitalization for the acute event as recoded in the patient’s health maintenance organization (HMO) file.

The study was conducted as part of the routine monitoring activities of the Ministry of Health and therefore did not require formal ethical approval.

Statistical analyses were performed using SPSS software for Windows version 19. Each patient was assigned to a group according to the initial rehabilitation services provided (inpatient, ambulatory only, none). Comparisons between the characteristics of patients were done by the chi- square test and t-test as appropriate. We conducted a multivariate logistic regression to explore the association between receipt of inpatient rehabilitation and the patient’s district of residence. Patients who were not mobile at baseline were excluded from the fracture analysis (9 patients).

The models included in addition to age the factors that were found to be linked to inpatient rehabilitation in a univariate analysis (p < 0.05): presence of dementia, dependency status and stroke severity.

For the purpose of sensitivity analysis we ran the regression models twice: once with dependent variable defined by the self reported data and once defined by the health provider's data.

## Results

Six one-day surveys were conducted between March, 2009 and October, 2010. 570 patients with stroke and 768 with hip fracture were identified, out of them 81 and 19 died in hospital and 489 and 749 were discharged and included in this study. The mean age of all the stroke patients was 78.8 years (range 65–100) and the mean age of all the hip fracture patients was 82.6 years (range 65–107). In-hospital mortality was much higher among stroke patients compared to fracture patients (14.2% versus 2.5%, respectively, *p* <0.001).

Interviews were held in 421/489 (86.1%) discharged stroke cases and 672/749 (89.9%) discharged fracture cases with the patients themselves (16.1%) or with their caretakers (83.9%). The distribution of rehabilitation setting by diagnosis is given in Table 1. 137 of all stroke patients (32.5%) and 82 (12.2%) of all the hip fracture patients did not receive any rehabilitation treatment. Stroke patients who did not receive any rehabilitation treatment were older and more often lived in a nursing home prior to the event. They were more often fully dependent prior to the hospitalization, had a diagnosis of dementia, and had undergone a more severe stroke (Table 2). Fracture patients who did not receive any rehabilitation were also more likely to be dependent prior to the event, to be diagnosed with dementia and to reside in a nursing home (Table 3). There was no age difference across the rehabilitation categories among the fracture patients.

**Table 1:** Distribution of first rehabilitation setting by diagnosis

	**Stroke**	**Hip fracture**
	**(N = 421)**	**(N = 672)**
	**N (%)**	**N (%)**
Inpatient followed by ambulatory	238 (56.5)	494 (73.5)
Ambulatory without inpatient	46 (10.9)	96 (14.3)
None	137 (32.5)	82 (12.2)

**Table 2:** Characteristics of stroke patients according to rehabilitation treatment setting

**Characteristic**	**Total**	**In-hospital**	**Ambulatory**	**No rehabilitation**	***P***
		**rehabilitation**	**rehabilitation**		
		**(N = 238)**	**(N = 46)**	**(N = 137)**	
Age (years)	78.3	77.3	78.4	79.9	0.007
Fully dependent at baseline	14.7	9.2%	20%	22.6%	<0.001
Mobile (Y/N)*	95.7%	97.6%	95.3%	92.6%	0.09
Dementia documented	11%	5.9%	8.7%	20.6%	<0.001
Living in a nursing home	4.3%	1.7%	0.0%	10.2%	<0.001
NIHSS mean	9.6	9.6	7.1	10.3	0.019
NIHSS median (range)	8	8 (1–25)	5 (1–26)	8 (1–31)	0.267

*Including mobility with assistance.

NIHSS, National Institutes of Health Stroke Scale.

**Table 3:** Characteristics of fracture patients according to rehabilitation treatment setting

**Characteristic**	**Total**	**In-hospital rehabilitation (N = 494)**	**Ambulatory rehabilitation (N = 96)**	**No rehabilitation (N = 82)**	***P***
Age (years)	82.6%	82.3	82.7	83.7	0.31
Fully dependent at baseline	14.9	9.5%	13.5%	48.8%	<0.001
Mobile (Y/N)* Dementia documented	98.5%	99.8%	98.8%	89.7%	<0.001
	16.1%	10.7%	17.7%	46.3%	<0.001
Living in a nursing home	3.6%	0.4%	1%	25.6%	0.001

*Including mobility with assistance.

Ambulatory rehabilitation treatments were given to 177/421 (42%) of the stroke patients and 432/672 (64%) of the fracture patients. Ambulatory treatments were given either as a sole treatment or mostly, following an episode of in-hospital rehabilitation both in stroke (131/177, 74%) and in fracture (336/432, 77%). However, a total of 107 (25%) stroke patients and 158 (24%) fracture patients did not receive any rehabilitation treatments in the community after discharge from inpatient rehabilitation.

In a multivariate analysis stroke patients residing in Jerusalem were two times less likely to receive inpatient rehabilitation compared to patients in the Centre District (OR 0.46, 95% CI 0.2-1.02, p = 0.057) (Table 4). Fracture patients residing in the districts with the lowest ratio of rehabilitation beds per elderly population (Jerusalem and the North) were 3–4 times less likely to receive inpatient rehabilitation compared to those living in the Centre district after controlling for age and baseline function (Table 5). Running the regression models with rehabilitation data based on the health provider‘s files gave similar results.

**Table 4:** Logistic Regression for inpatient rehabilitation – stroke

**Variable**	**Interview data (N = 404)**			**Health provider’s data (N = 459)**		
	**OR**	***P***	**95% confidence interval**	**OR**	***P***	**95% confidence interval**
Age	0.97	0.036	0.94-0.99	0.96	0.009	0.94-0.99
Dependent at baseline	0.51	0.075	0.25-1.07	0.44	0.03	0.21-0.92
Dementia at baseline	0.32	0.013	0.14-0.8	0.38	0.019	0.17-0.85
NIHSS		<0.001			<0.001	
5-24	1	NA	NA	1	NA	NA
<=4	0.28	<0.001	0.17-0.46	0.28	<0.001	0.17-0.46
>24	0.07	0.014	0.01-0.59	0.07	0.014	0.01-0.59
District*		0.25			0.07	
Center	1	NA		1	NA	
Haifa	1.12	0.75	0.55-2.3	1.01	0.98	0.53-1.9
Tel Aviv	0.97	0.93	0.51-1.85	1.19	0.56	0.66-2.15
South	0.56	0.15	0.25-1.23	0.81	0.58	0.39-1.69
Jerusalem	0.46	0.057	0.21-1.02	0.38	0.014	0.18-0.82
North	0.81	0.554	0.41-1.61	0.68	0.213	0.36-1.25

The following categories were used as the reference: not being dependent at baseline, absence of dementia, NIHSS of 5–24, residence in the Center district.

* Districts were ranked according to availability of rehabilitation beds ranging from 4 to 0.5 per 1000 population ages 65 and above.

**Table 5:** Logistic regression for inpatient rehabilitation – fracture

**Variable**	Interview data (N = 588)*			Health provider’s data (N = 459)*		
	**OR**	***P***	**95% confidence interval**	**OR**	***P***	**95% confidence interval**
Age	0.99	0.36	0.96-1.02	0.99	0.3	0.96-1.01
Dependent at baseline	0.4	0.002	0.22-0.72	0.48	0.009	0.27-0.83
Dementia at baseline	0.47	0.011	0.26-0.84	0.41	0.001	0.24-0.7
District^†^		<0.001			<0.001	
Center	1	NA		1	NA	
Haifa	0.78	0.484	0.4-1.55	0.62	0.115	0.34-1.13
Tel Aviv	1.02	0.95	0.58-1.8	0.91	0.707	0.55-1.51
South	0.72	0.5	0.28-1.88	1.19	0.708	0.48-2.97
Jerusalem	0.23	<0.001	0.12-0.44	0.24	<0.001	0.12-0.48
North	0.29	0.001	0.22-0.72	0.48	0.009	0.27-0.83

The following categories were used as the reference: not being dependent at baseline, absence of dementia, residence in the Center district.

* Patients who were not mobile at baseline were excluded.

† Districts were ranked according to availability of rehabilitation beds ranging from 4 to 0.5 per 1000 population ages 65 and above.

## Discussion

We report the results of the first national study to assess the extent of rehabilitation services provided to the elderly following an event of acute stroke or hip fracture in Israel. The vast majority of patients received some form of rehabilitation treatment, mostly within specialized rehabilitation wards (56.5% of the stroke patients and 73.5% of the fracture patients were referred to inpatient rehabilitation services). These rates are similar to the 52.2% inpatient rehabilitation rate reported from a stroke registry in Germany [18] but higher than the 29% reported from Ontario stroke registry [19], though these rates refer to all adult stroke patients and not just the elderly as in our work. In regards to fracture, in Scotland and the UK fewer than 50% of the patients were discharged to a rehabilitation unit from the acute care [20,21] but in these countries the model of care is different with integration of orthogeriatric and multidisciplinary review into the acute care [10,11]. It should be noted, that international comparisons of inpatient rehabilitation rates are problematic because of differences between health systems, the structure of rehabilitation services and the means of funding. In countries such as the UK, Canada, Australia and New Zealand, there are programs for early supported discharge, that provide a service of organized multidisciplinary and intensive rehabilitation treatments at home, as an alternative for inpatient rehabilitation for suitable patients with mild to moderate disability [10,22,23]. Inpatient rehabilitation in the USA can be given either in rehabilitation hospitals or within specialized nursing facilities [24]. Our findings revealed that rehabilitation after stroke and hip fracture in Israel is provided mainly through inpatient settings.

In the present study, almost one-third of the stroke patients and 12.2% of the fracture patients received no rehabilitation treatment in any setting. As might be expected, these patients were, on the average, older and more likely to be dependent and bed-bound before the event. Yet, the majority of them lived in the community prior to the event. In addition, 50% of the stroke patients and 30% of the fracture patients that did not get any rehabilitation were discharged to their homes and not to a geriatric nursing facility, suggesting that this group might include individuals that could have benefited from a trial of rehabilitation.

Selecting people for rehabilitation poses a difficult dilemma in trying to balance between the objective to provide proper treatment for all the patients that may benefit from it, and the need to avoid wasting scarce resources on candidates who are expected to fail [25,26]. Available models that aim to predict rehabilitation outcomes in order to assist clinicians in selecting the appropriate patients for rehabilitation have high positive predictive values (i.e., those who were predicted to benefit from rehabilitation indeed regained functional ability) but low negative predictive values [27-29]. Therefore, using these models as a selection tool will lead to denying treatment from many patients who may benefit from it. Given the lack of efficient objective tools, the process of selecting patients for rehabilitation becomes a matter of combining clinical judgment with various nonclinical considerations, such as the distance of the patient’s home from the rehabilitation facility, the type of admitting ward (e.g., orthopedic or geriatric ward) and the patient’s socio-demographic factors. The influence of these non-clinical factors on the decision-making process can lead to inequities in access to rehabilitation [25,30].

The geographic disparities in rehabilitation rates that were observed in this study lend support to the premise that non-patient-related factors play a role in the selection process. Geographic disparities in rehabilitation care were reported in the USA both between states and between regions in the same state [31]. Freburger et al. [30] found that post-acute care varied significantly between four states in the USA even after controlling for patient and hospital characteristics. A study performed within the Northern California Kaiser Permanente Health System found that patients in one administrative area were twice more likely to get inpatient rehabilitation than patients in other areas [32].

Supply could be one reason for geographic variation, since the availability of post-acute care services was found to strongly limit their appropriate use following an acute event of hip fracture, stroke or joint replacement [33]. We detected lower inpatient rehabilitation rates in the North and the Jerusalem districts which were noted in the annual report of the State Comptroller for 2011 to have a low ratio of rehabilitation beds per the elderly population [34] Compared to the national average of one rehabilitation bed per ~500 elderly citizens, the ratios were 1 per 1268 and 1 per 3200 in the Jerusalem and the North districts, respectively. Our findings are also supported by an earlier work of the NASIS team in Israel that reported a lower inpatient rehabilitation rate for stroke patients in the North district in 2008, and attributed it to a shortage in rehabilitation beds [35].

The availability of beds is a pre-condition for the provision of rehabilitation services, but it does not guarantee an equal access to those services. In practice, the provision of inpatient rehabilitation care is a multistage process that involves the interaction of many factors involved in the patient‘s care. The acute care ward needs to assess the need and potential for rehabilitation and refer the patient for further treatment, the healthcare provider must agree to pay for the additional treatments, the rehabilitation unit needs to accept the patient and, finally, the patient and his/her family need to agree to the suggested rehabilitation program. In order to streamline the referral process and ensure the patient’s right to receive proper rehabilitation care, the Ministry of Health Geriatric Department issued a circular with practice guidelines for rehabilitation therapy for the elderly in 2009 [15]. These guidelines specified the medical conditions for which rehabilitation care is indicated and defined the responsibilities of each of the professional bodies involved in the care process. Variation in protocols between hospital wards and in the implementation of these guidelines could have contributed to the regional disparities that were detected in our survey. Wolfe et al.’s study that compared patterns of stroke care between 11 hospitals in seven European countries found a large variation in the use of rehabilitation treatment [36]. The lack of clear criteria to define ”suitability for rehabilitation“ leads to large variations in the selection of people for rehabilitation, both by the acute referring ward as well as by the rehabilitation service. An Australian study that compared the distribution of discharge destinations between seven acute stroke units found that the proportion of severe stroke cases discharged to a rehabilitation facility varied as widely as 27% to 70% [25]. In a clinical audit in Australia, the proportion of stroke patients discharged to inpatient rehabilitation was higher in stroke units and in units with larger volumes of patients (at least 100 stroke admissions per year) [37]. The specialization of the health professional that assesses the patient‘s suitability for rehabilitation care also plays an important role. An Australian single-center study reported that the acute care team found patients ready for transfer to rehabilitation 2.5 days earlier on average, compared to the assessment of the rehabilitation team, and only 75% of the patients referred by the acute team were accepted by the rehabilitation team [38]. Although the practice guidelines issued by the Israeli Ministry of Health state that the rehabilitation potential should be assessed by a specialist in either rehabilitation medicine or in geriatrics, this is often not implemented in practice. One of the obstacles is the fact that some hospitals do not employ such a specialist on a full-time basis [35].

In our data we did not detect differences in the rates of inpatient rehabilitation between types of wards but the frequency of including treatment recommendations regarding rehabilitation in the discharge letter differed: The issue of rehabilitation was not mentioned in the discharge letter of 35.6% of the fracture patients discharged from the orthopedic ward compared to only 12.5% of those discharged from the geriatric ward (p < 0.001).

The present study is the first national survey that assessed the extent of rehabilitation services provided to the elderly in Israel. By including all 26 acute care hospitals in Israel, it provides a representative national overview. The usage of different resources increased the validity and completeness of the collected data. We are aware that there was a great variability in the quality of discharge letters from different hospitals, resulting in some lack of information on the functional status of the patient before the event and at discharge. Decisions with regard to rehabilitation therapy were not always well documented in the discharge letters so we could not always distinguish between patients who were assessed clinically to be not fit for rehabilitation versus patients who did not receive rehabilitation for other reasons. This limitation was valid for all districts and therefore could not explain the regional gaps that were documented in our study. The use of proxy respondents of different relatedness to the patient could theoretically impair the validity of the data. But as the respondents were closely involved with the patient care we believe the validity was quite high as demonstrated by the good correlation with the health-provider’s data.

On the national level, policy makers in the Ministry of Health should initiate appropriate planning of rehabilitation services based on the current and future needs of the growing population. Such planning should consider the growth and aging of the population in each district over the next 10 years, the incidence of medical conditions that require rehabilitation and the estimated mean number of inpatient rehabilitation days per event. If the planned reform to integrate nursing services with primary care services goes through, this may encourage the Health Maintenance Organizations to provide rehabilitation services. In addition, there should be ongoing supervision of the implementation of the guidelines set by the Ministry of Health.

## Conclusion

In conclusion, distinct regional differences were found in the provision of rehabilitation care after an acute episode of stroke or hip fracture, and these differences seemed to be related to the availability of rehabilitation beds. An ongoing follow-up survey will examine whether this trend continues and attempt to better characterize its causes.

## Competing interest

The authors declare that they have no competing interests.

## Authors’ contribution

All the authors were actively involved in designing the study. IZ - Data analysis and manuscript writing. TS – Data analysis and revision of the manuscript. IL, IR, SH and AC - revision of the manuscript. All authors read and approved the final manuscript.

## References

[B1] Stucki G Stier-Jarmer M Grill E Melvin J Rationale and principles of early rehabilitations care after an acute injury or illness Disabil Rehabil 2005 27 7/8 353 359 16040536 10.1080/09638280400014105

[B2] Feigin VL Lawes CMM Bennett DA Anderson CS Stroke epidemiology: A review of population-based studies of incidence, prevalence, and case-fatality in the late 20th century Lancet Neurol 2003 2 1 43 45 10.1016/S1474-4422(03)00266-7 12849300

[B3] Johnel O Gullberg B Allander E Kanis J The apparent incidence of hip fracture in europe: A study of national register sources Osteoporosis Int 1992 2 6 298 302 10.1007/BF01623186 1421798

[B4] Dixon T Shaw M Ebrahim S Dieppe P Trends in hip and knee joint replacement: Socioeconomic inequalities and projections of need Ann Rheum Dis 2004 63 7 825 830 10.1136/ard.2003.012724 15194578 PMC1755069

[B5] Covinsky KE Palmer RM Fortinsky RH Counsell SR Stewart AL Kresevic D Burant CJ Landefeld CS Loss of independence in activities of daily living in older adults hospitalized with medical illnesses: Increased vulnerability with age J Am Geriatr Soc 2003 51 4 451 458 10.1046/j.1532-5415.2003.51152.x 12657063

[B6] Chudyk AM Jutai JW Petrella RJ Speechley M Systematic review of hip fracture rehabilitation practices in the elderly Arch Phys Med Rehabil 2009 90 2 246 262 10.1016/j.apmr.2008.06.036 19236978

[B7] Quinn TJ Paolucci S Sunnerhagen KS Sivenius J Walker MF Toni D Lees KR European Stroke Organisation (ESO) Executive Committee; ESO Writing Committee Evidence-based stroke rehabilitation: An expanded guidance document from the European stroke organization (ESO) guidelines for management of ischaemic stroke and transient ischaemic attack 2008 J Rehabil Med 2009 41 2 99 111 10.2340/16501977-0301 19225703

[B8] Duncan PW Horner RD Reker DM Samsa GP Hoenig H Hamilton B LaClair BJ Dudley TK Adherence to postacute rehabilitation guidelines is associated with functional recovery in stroke Stroke 2002 33 1 167 178 10.1161/hs0102.101014 11779907

[B9] Halbert J Crotty M Whitehead C Cameron I Kurrle S Graham S Handoll H Finnegan T Jones T Foley A Shanahan M Hip Fracture Rehabilitation Trial Collaborative Group Multi-disciplinary rehabilitation after hip fracture is associated with improved outcome: A systematic review J Rehabil Med 2007 39 7 507 512 10.2340/16501977-0102 17724548

[B10] National Institute for Health and Clinical Excellence The management of hip fracture in adults 2011 London: NICE Clinical Guidelines 124 http://www.nice.org.uk/CG124

[B11] Scottish Intercollegiate Guidelines Network Prevention and management of hip fracture in older people 2009 111 http://www.sign.ac.uk/guidelines/fulltext/111/index.html

[B12] Mak JCS Cameron ID March LM Evidence-based guidelines for the management of hip fractures in older persons: an update Med J Aust 2010 192 1 37 41 20047547 10.5694/j.1326-5377.2010.tb03400.x

[B13] Miller EL Murray L Richards L Zorowitz RD Bakas T Clark P Billinger SA American Heart Association Council on Cardiovascular Nursing and the Stroke Council: comprehensive overview of nursing and interdisciplinary rehabilitation care of the stroke patient Stroke 2010 41 10 2402 2448 10.1161/STR.0b013e3181e7512b 20813995

[B14] Lindsay P Bayley M Chelsea Hellings BSH Hill M Woodbury E Phillips S Canadian Stroke Strategy Best Practices and Standards Writing Group on behalf of the Canadian Stroke Strategy, a joint initiative of the Canadian Stroke Network and the Heart and Stroke Foundation of Canada): Canadian best practice recommendations for stroke care (updated 2008) Can Med Assoc J 2008 179 12 S1 S25 10.1503/cmaj.081148.R2 PMC2585121 19047599

[B15] Medical Administration, Ministry of Health Criteria for granting rehabilitative care for elderly 2009 http://www.health.gov.il/hozer/mr04_2009.pdf

[B16] Kasner SE Chalela JA Luciano JM Cucchiara BL Raps EC McGarvey ML Conroy MB Localio AR Reliability and validity of estimating the NIH stroke scale score from medical records Stroke 1999 30 8 1534 1537 10.1161/01.STR.30.8.1534 10436096

[B17] Schlegel D Kolb SJ Luciano JM Tovar JM Cucchiara BL Liebedkind DS Kasner SE Utility of the NIH Stroke Scale as a Predictor of Hospital Disposition Stroke 2003 34 1 134 137 10.1161/01.STR.0000048217.44714.02 12511764

[B18] Unrath M Kalic M Berger K Who receives rehabilitation after stroke? Data from the quality assurance project “Stroke Register Northwest Germany” Deutsches Ärzteblatt International 2013 110 7 101 107 23468819 10.3238/arztebl.2013.0101 PMC3585451

[B19] Canadian Institute for Health Information Pathways of Care for People with Stroke in Ontario 2012 https://secure.cihi.ca/free_products/Pathways_of_care_aib_en.pdf

[B20] National Services Scotland (NHS) The patient journey post hip fracture: What constitutes rehabilitation? A Report from the Scottish Hip Fracture Audit 2009 http://www.shfa.scot.nhs.uk/Rehab_Report_2009.pdf

[B21] National Hip Fracture Database (NHFD) National report 2012 2012 http://www.nhfd.co.uk/

[B22] National Stroke Foundation (Australia) Clinical guidelines for stroke management 2010. National Stroke Foundation 2010 http://www.strokefoundation.com.au/index2.php?option=com_docman&task=doc_view&gid=329&Itemid=39

[B23] New Zealand Guidelines Group Acute management and immediate rehabilitation after hip fracture amongst people aged 65 years and over 2003 http://www.nzgg.org.nz/library_resources/6_hip_fracture_management_guideline

[B24] Buntin MB Colla CH Deb P Sood N Escarce JJ Medicare spending and outcomes after postacute care for stroke and hip fracture Med Care 2010 48 9 776 784 10.1097/MLR.0b013e3181e359df 20706167 PMC3627731

[B25] Ilett PA Brock KA Graven CJ Cotton SM Selecting patients for rehabilitation after acute stroke: Are there variations in practice? Arch Phys Med Rehabil 2010 91 5 788 793 10.1016/j.apmr.2009.11.028 20434618

[B26] Wade DT Selection criteria for rehabilitation services Clin Rehabil 2003 17 2 115 118 10.1191/0269215503cr591ed 12625650

[B27] Kollen B Kwakkel G Lindeman E Longitudinal robustness of variables predicting independent gait following severe middle cerebral artery stroke: A prospective cohort study Clin Rehabil 2006 20 3 262 268 10.1191/0269215506cr910oa 16634346

[B28] Wilson D Houle D Keith R Stroke rehabilitation: A model predicting return home West J Med 1991 154 5 587 590 1866956 PMC1002838

[B29] Brauer SG Bew PG Kuys SS Lynch MR Morrison G Prediction of discharge destination after stroke using the motor assessment scale on admission: A prospective, multisite study Arch Phys Med Rehabil 2008 89 6 1061 1065 10.1016/j.apmr.2007.10.042 18503800

[B30] Freburger JK Holmes GM Ku LJE Cutchin MP Heatwole-Shank K Edwards LJ Disparities in postacute rehabilitation care for stroke: An analysis of the state inpatient databases Arch Phys Med Rehabil 2011 92 8 1220 1229 10.1016/j.apmr.2011.03.019 21807141 PMC4332528

[B31] Buntin MB Access to postacute rehabilitation Arch Phys Med Rehabil 2007 88 11 1488 1493 10.1016/j.apmr.2007.07.023 17964894

[B32] Sandel ME Wang H Terdiman J Hoffman JM Ciol MA Sidney S Quesenberry C Lu Q Chan L Disparities in stroke rehabilitation: Results of a study in an integrated health system in northern California PM R 2009 1 1 29 40 10.1016/j.pmrj.2008.10.012 19627870 PMC3432287

[B33] Buntin MB Garten AD Paddock S Saliba D Totten M Escarce JJ How much is postacute care use affected by its availability? Health Serv Res 2005 40 2 413 434 10.1111/j.1475-6773.2005.0i366.x 15762900 PMC1361149

[B34] State Comptroller 62 annual audit report for 2011 and fiscal 2010 accounts 2012 http://www.mevaker.gov.il/serve/contentTree.asp?bookid=611&id=57&contentid=&parentcid=undefined&sw=1280&hw=954

[B35] Treger I Ring H Schwartz R Tsabari R Bornstein NM Tanne D Hospital disposition after stroke in a national survey of acute cerebrovascular diseases in Israel Arch Phys Med Rehabil 2008 89 3 435 440 10.1016/j.apmr.2007.11.001 18295620

[B36] Wolfe C Tilling K Rudd A Giroud M Inzitari D Variations in care and outcome in the first year after stroke: A western and central European perspective J Neurol NeurosurgPsychiatry 2004 75 12 1702 1706 10.1136/jnnp.2004.039438 PMC1738847 15548486

[B37] National Stroke Audit Acute Services National Stroke Audit Acute Services: Clinical Audit Report 2011. National Stroke Foundation. Accessed from 2011 http://strokefoundation.com.au/site/media/National_stroke_audit_acute_services_clinical_audit_report_2011.pdf

[B38] Poulos CJ Magee C Bashford G Eagar K Determining level of care appropriateness in the patient journey from acute care to rehabilitation BMC Health Serv Res 2011 11 1 291 10.1186/1472-6963-11-291 22040281 PMC3212985

